# Early Postoperative Mortality Risk Factors and Five- and Ten-Year Mortality Rates After Hip Arthroplasty for Femoral Neck Fracture

**DOI:** 10.3390/jcm14238263

**Published:** 2025-11-21

**Authors:** Khalil Khalil, Youssef Jamaleddine, Ahmad Haj Hussein, Elio Daccache, Joseph Mouawad, Guillaume Fricault, Alfred Khoury, Nicolas Reina

**Affiliations:** 1 Lebanese American University Medical Center-Rizk Hospital, Beirut 11-3288, Lebanon; khaliltkhalil@hotmail.com (K.K.); ahmad.hajhussein@lau.edu (A.H.H.); elio.daccache1@gmail.com (E.D.); joeamouawad@gmail.com (J.M.); fred_khoury@yahoo.com (A.K.); 2 Hôpital Pierre Paul Riquet, CHU Toulouse, Place du Dr Baylac, 31300 Toulouse, France; fricault.guillaume@gmail.com (G.F.); reina.n@chu-toulouse.fr (N.R.)

**Keywords:** femoral neck fracture, arthroplasty, mortality, hemiarthroplasty, total hip arthroplasty, hip

## Abstract

**Introduction:** Femoral neck fractures in older adults are associated with appreciable short-term mortality, yet long-term survival after hip arthroplasty is incompletely characterized. We analyzed early mortality risk factors and 5- and 10-year mortality after hemi-arthroplasty or total hip arthroplasty (THA) for femoral neck fractures. **Materials and Methods:** In this single-center retrospective cohort, 397 consecutive patients underwent arthroplasty for femoral neck fracture in 2014 and 2015. Mean age was 83.3 years and 70.3% were women. Demographic data, Charlson Comorbidity Index, Parker Mobility Score, medication history, operative and anesthetic details, transfusion, and peri-operative complications were extracted. Survival status up to 10 years was obtained from hospital and civil registries. *p*-value < 0.05 was considered statistically significant. **Results:** A total of 397 patients were included. When categorized by age and ASA scores into low-, medium-, and high-risk groups, mortality rates increased with higher risk (*p* < 0.001). The mortality rate at 30 days, 90 days, 1 year, 5 years, and 10 years postoperatively was 3.5%, 7.1%, 14.1%, 48.36% and 71.03%, respectively; mean time-to-death was 3.3 years. At 30 days, mortality was higher in males, those on clopidogrel, in patients with lower mobility (lower Parker Score), higher morbidity (higher Charlson Score), NNIS score of 1, higher ASA, patient who underwent hemiarthroplasty, and patients with medical complications post-op. Additional 90-day risks were antivitamin K therapy, immunosuppressants, and continuous spinal anesthesia; 1-year risks also encompassed advanced age, prolonged hospital stay, and peri-operative transfusion. **Conclusions:** Arthroplasty after femoral neck fracture is associated with high mortality rate; only half of patients survive 5 years and fewer than one-third reach 10 years. Mortality rate is affected by many risk factors, both non-modifiable factors and modifiable peri-operative variables. Targeted optimization of modifiable peri-operative factors and multidisciplinary geriatric-orthopedic care may improve outcomes in this frail population.

## 1. Introduction

Hip fractures are a major global health problem. Current estimates suggest 1.6–2 million cases occur each year, and this number is projected to rise to more than 6 million annually by 2050 as the population ages [1]. Data from the Global Burden of Disease 2019 study reported an incidence of 681 per 100,000 persons aged ≥ 55 years, showing a steady increase in case numbers. While some regions have seen stable or falling age-adjusted rates, international studies confirm that the absolute number of hip fractures will almost double by mid-century, creating a growing challenge for healthcare systems worldwide [1,2]. These fractures are associated with profound morbidity and remain among the leading causes of disability and loss of independence in the geriatric population [3]. Surgical arthroplasty—either hemiarthroplasty (HA) or total hip arthroplasty (THA)—has become the preferred management for displaced femoral neck fractures, offering pain relief, rapid mobilization, and reduced complication rates compared to fixation. Recent analyses highlight that while both HA and THA are increasingly utilized, THA is rising at a faster rate, reflecting an effort to improve long-term functional outcomes [4]. The modeling study on hip fracture rates in Romania showed that an increase in THA prevalence contributed to an increase in expected hip fracture cases [5].

Despite these advances, mortality following femoral neck fracture remains alarmingly high. Early postoperative mortality ranges between 5–10% at 30 days, and 1-year mortality is consistently reported between 20% and 30% [6,7]. Long-term outcomes are similarly concerning: registry-based studies have shown excess mortality persists for years after surgery, with standardized mortality ratios remaining nearly 2.5 times higher than in the general population even up to six years post-fracture [1]. These data emphasize that femoral neck fractures are not isolated orthopedic events, but rather sentinel markers of frailty and vulnerability in elderly patients.

To improve risk stratification, several prognostic tools have been validated in recent years. The American Society of Anesthesiologists (ASA) score and the Charlson Comorbidity Index (CCI) are widely adopted to quantify perioperative risk and comorbidity burden, with higher scores strongly correlating with increased short- and long-term mortality [8]. Similarly, pre-fracture mobility status, often measured by the Parker Mobility Score (or New Mobility Score), has been shown to be a powerful predictor of both functional recovery and survival [9]. Patients with limited mobility prior to injury have significantly poorer outcomes compared to those with higher baseline functional independence.

Distinguishing between modifiable and non-modifiable risk factors is of particular importance. Non-modifiable determinants include advanced age, male sex, and preexisting comorbidities, all of which consistently predict higher mortality [8]. On the other hand, modifiable factors, such as perioperative optimization of comorbidities, timely surgery, transfusion practices, type of anesthesia, and prevention of postoperative complications, represent opportunities for clinicians to improve outcomes. Recent literature has also highlighted the benefits of orthogeriatric co-management and multidisciplinary perioperative care pathways in reducing complications and mortality [9].

Given the rising global incidence of femoral neck fractures, the increasing reliance on hip arthroplasty, and the persistently high mortality rates, there is a pressing need to better characterize both early and long-term outcomes. This study therefore aims to evaluate predictors of mortality in patients undergoing HA or THA for femoral neck fractures at a single institution, analyzing clinical, surgical, and functional variables over a ten-year follow-up. By identifying key prognostic indicators, we aim to refine risk stratification and highlight the importance of targeting modifiable perioperative factors within multidisciplinary care strategies.

## 2. Materials and Methods

### 2.1. Study Design and Population

**Figure 1:**
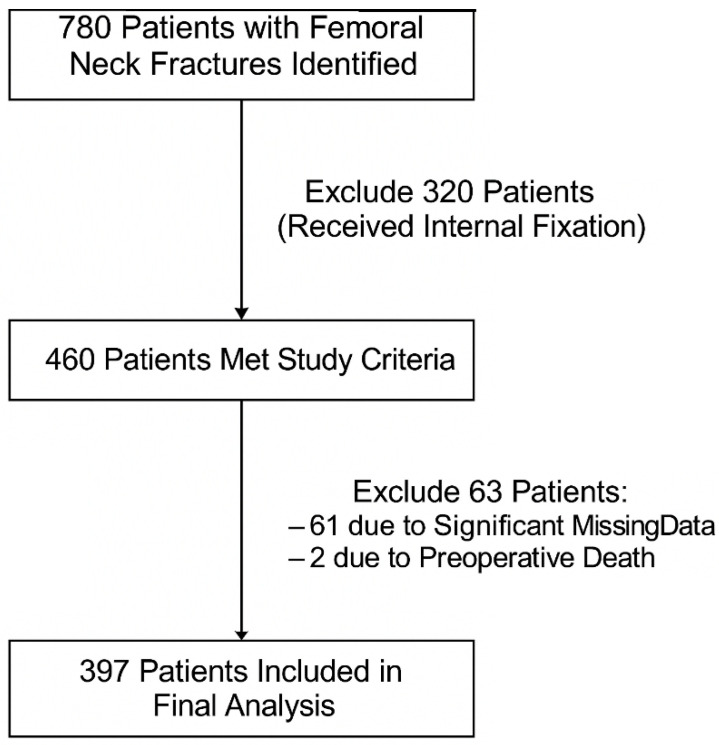
Flow diagram of study population inclusion and exclusion.

This retrospective study included 397 patients diagnosed with femoral neck fractures and treated by arthroplasty in a single academic center in France, in 2014 and 2015. Inclusion criteria were limited to patients who underwent either hemiarthroplasty or total hip arthroplasty for femoral neck fractures. Exclusion criteria included any patients that were treated with a different surgical technique, including internal fixation using screws or dynamic hip screws, as well as those who did not have surgery because of medical comorbidities that prohibited the procedure. A flowchart illustrating the patient inclusion and exclusion is presented in Figure 1.

The primary outcome of this study was the cumulative mortality rate at 1-, 3- and 12-months post-fracture, and subsequent potential risk factors and at 5-year and 10-year interval for calculation of survival rate.

To assess risk factors, several factors were implemented:

Demographics and function (age, gender, body mass index (BMI), comorbidities, smoking status, nutritional status, medication history, and Charlson Comorbidity Index [10] and Parker Score [11]);

Intraoperative details (Surgical approach, type of arthroplasty, type of fixation, and type of anesthesia);

Perioperative factors (transfusion, time to surgery, hospital stay).

Mortality was obtained using the institution registry and the French civil registry for patients lost to follow-up, ensuring accurate data collection.

### 2.2. Statistical Methods

The normality of the data was assessed using the Shapiro test. Continuous variables were compared between mortality groups using *t*-test and Mann–Whitney U test as appropriate. Chi-square or Fisher’s exact were used as appropriate for the bivariate analyses of the categorical variables. The correlation between two quantitative variables was assessed using the Pearson correlation test. Multivariable Cox proportional hazards regression was used to predict mortality after 1 year post op. *p*-value < 0.05 was considered statistically significant.

All statistical analyses were performed using IBM SPSS Statistics for Windows, Version 27.0 (IBM Corp., Armonk, NY, USA).

## 3. Results

### 3.1. Sample and Surgical Characteristics

The mean age of the study population was 83.3 years. The majority of patients were female (70.3%). The mean BMI was 23.09. Patients were current smokers in 10.9%. Surgical characteristics are shown in Table 1 and Table 2. Pre-op complications are described in Table 3. Mortality rates were calculated at 30 days, 90 days, and 1 year postoperatively.

### 3.2. Post-Op Medical and Surgical Complications

A total of 55 patients experienced a post op medical complication (Table 4), and 44 experienced a post op surgical complication (Table 5).

### 3.3. Mortality Rate

The mortality rate at 30 days was 3.5% (14 patients). The mortality rate at 90 days increased to 7.1% (28 patients). At 1 year postoperatively, the mortality rate increased to 14.1% (56 patients).

At 5 years follow-up, 205 individuals were alive, yielding a survival rate of 51.6%. At 10 years follow-up, 115 individuals remained alive, resulting in a survival rate of 29%. The average time to death was 3.3 years.

**Figure 2:**
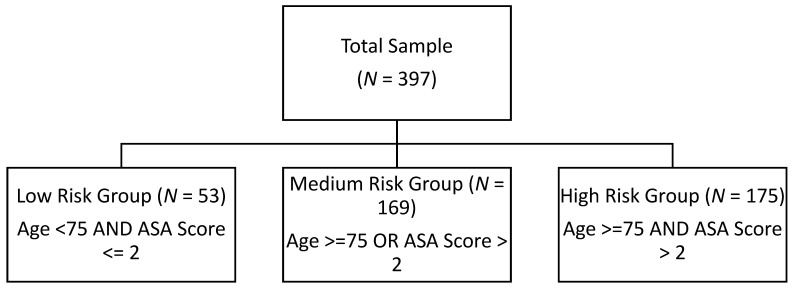
Stratification of population into three risk groups based on age and ASA.

The population was stratified into three risk groups based on age and ASA (American Society of Anesthesiologists) scores (Figure 2). The low-risk group (N = 53) included patients < 75 years with an ASA score ≤ 2. The medium-risk group (N = 169) consisted of patients who were either aged > 75 or an ASA score > 2. The high-risk group (N = 175) comprised patients aged > 75 with an ASA score > 2.

The differences in mortality rates across these groups were statistically significant (Table 6), with *p*-values <0.001. The low-risk group had 0% mortality at all time points (30 days, 90 days, 1 year). The medium-risk group showed increasing mortality: 0.6% at 30 days, 3% at 90 days, and 10.7% at 1 year. The high-risk group had substantially higher rates: 7.4% at 30 days, 13.1% at 90 days, and 21.7% at 1 year.

**Mortality rates at 30 days, 90 days, and 1 year (Table 7).**

**30-Day Mortality:**

Gender was a significant factor with higher mortality in males; 7.6% versus 1.8%, respectively (*p* = 0.007). Patients on immunosuppressants had higher mortality (11.5% vs. 3%, *p* = 0.057), similarly patient on Clopidogrel had higher risk of mortality compared to patients who did not take it (15.8% vs. 2.7% respectively, *p* = 0.021). The Parker Mobility Score and Total Charlson Score were lower and higher, respectively, in deceased patients, indicating reduced mobility and higher comorbidity as risk factors for higher mortality (Parker Score: *p* = 0.04, Charlson Score: *p* = 0.004). Additionally, patients ASA 3 (5.9%) or 4 (15.8%) have greater mortality than ASA score 1 (0%) and 2 (0.6%) *p* < 0.001. Patients with NNIS score of 1 had significantly higher mortality (*p* = 0.002). Those undergoing hemiarthroplasty have higher mortality than those undergoing total hip arthroplasty (5.1% vs. 0.7% respectively, *p* = 0.024). Postoperative medical complications were strongly associated with increased mortality compared to those who did not have complications (12.3% vs. 1.8% respectively, *p* < 0.001).

Other factors were not associated with increased mortality at 30 days.

**90-Day Mortality:**

Gender is still a risk factor for mortality, with males having a higher mortality rate (11% vs. 5.4% in females, *p* = 0.045). Patients on immunosuppressants had higher mortality (19.2%) compared to those not on such medication (6.2%, *p* = 0.029). Clopidogrel usage was a significant factor (*p* = 0.035), with patients on this medication showing a 21.1% mortality rate, much higher than those not on Clopidogrel (6.2%). Patients on antivitamin K medications also had higher mortality than those who are not on it (11.5% vs. 5.6% respectively, *p* = 0.043). Mortality was associated with a lower Parker Mobility Score and a higher Charlson Score (*p* = 0.009 and *p* = 0.021, respectively). Higher ASA scores further indicated increased mortality risk (ASA 3: 12.4%, ASA 4: 21.1%, *p* < 0.001). Patients with NNIS score of 1 had higher mortality rates at 13.3% (*p* < 0.001). Continuous spinal anesthesia presented a higher mortality risk (26.1%) compared to other anesthesia types (*p* = 0.028), and patients experiencing medical complications postoperatively had a 15.4% mortality rate versus 5.4% in those without complications (*p* = 0.013). Those undergoing hemiarthroplasty had higher mortality than those undergoing total hip arthroplasty (9.7% vs. 2.1% respectively, *p* = 0.005).

Other factors were not associated with increased mortality at 90 days.

**1-Year Mortality:**

Age became a significant risk factor, with deceased patients having a mean age of 86.9 years compared to 82.7 in survivors (*p* = 0.006). Male mortality remained higher in males then females (22% vs. 10.8%, *p* = 0.003), and at 1 year the prolonged hospital stays were linked with increased mortality (11.21 vs. 9.275 days, *p* = 0.038). Clopidogrel and antivitamin K use continued to be associated with higher mortality (Clopidogrel: 31.6% vs. 13%, *p* = 0.035; antivitamin K: 20.2% vs. 12.2%, *p* = 0.046), as did immunosuppressant use (34.6% vs. 12.7%, *p* = 0.006). Lower Parker Mobility and higher Charlson Scores remained significant risk factors for mortality (*p* < 0.001 for both), and patients who received pRBC transfusions had a higher mortality rate (23.9% vs. 10.2%, *p* < 0.001). Patients with NNIS score of 1 had higher mortality rates at 22.3% (*p* < 0.001). Those undergoing hemiarthroplasty had higher mortality than those undergoing total hip arthroplasty (18.7% vs. 5.7% respectively, *p* < 0.001). Continuous spinal anesthesia had the highest 1-year mortality rate (39.1%, *p* = 0.011), and postoperative medical complications were again a strong predictor, with a mortality rate of 26.2% among affected patients (*p* = 0.002).

Other factors were not associated with increased mortality at 1 year.

**Bivariate Correlations (Table 8).**

For 30-day mortality, both the Charlson Comorbidity Index (r = 0.142, *p* < 0.01) and the ASA score (r = 0.187, *p* < 0.001) were positively correlated, indicating that patients with higher Charlson and ASA scores had a higher likelihood of 30-day mortality.

At 90 days, a significant positive correlation was observed between hospitalization duration and mortality (r = 0.109, *p* < 0.05), as well as between the Charlson Comorbidity Index (r = 0.116, *p* < 0.05) and ASA score (r = 0.229, *p* < 0.001). Additionally, the Parker Mobility Score was inversely correlated with mortality (r = −0.112, *p* < 0.05), suggesting that patients with greater mobility had lower 90-day mortality risk.

At 1-year mortality, age was positively correlated with 1-year mortality (r = 0.140, *p* < 0.01), as were hospitalization duration (r = 0.105, *p* < 0.05), Charlson Comorbidity Index (r = 0.256, *p* < 0.001), and ASA score (r = 0.249, *p* < 0.001). The Parker Mobility Score was again negatively correlated (r = −0.190, *p* < 0.001), indicating that patients with higher mobility scores experienced reduced mortality at one year.

**Multivariate Cox proportional hazards regression model** (Table 9).

In the multivariable Cox proportional hazards regression (Omnibus test: χ^2^ = 52.3, df = 8, *p* < 0.001), several predictors were significantly associated with 1-year mortality following hip arthroplasty.

Lower ASA classification was associated with reduced survival compared with ASA IV. Patients with ASA II and ASA III had significantly lower hazards of mortality, with HR = 0.30 (95% CI: 0.11–0.81, *p* = 0.017) and HR = 0.40 (95% CI: 0.17–0.95, *p* = 0.038), respectively. Estimates for ASA I were unstable due to the relatively small number of patients in this category (HR = 0.00, 95% CI: 0.00–3.04 × 10^3^, *p* = 0.970).

The Charlson Comorbidity Index was independently predictive of mortality, with each unit increase associated with a 23% higher hazard (HR = 1.23, 95% CI: 1.04–1.47, *p* = 0.018). Preoperative Parker Mobility Score was protective, with higher functional status linked to lower risk of death (HR = 0.86, 95% CI: 0.76–0.97, *p* = 0.014). The strongest predictor was postoperative medical complications, which were associated with nearly a three-fold increase in 1-year mortality (HR = 2.88, 95% CI: 1.56–5.34, *p* < 0.001).

Age and gender demonstrated hazard ratios consistent with higher mortality risk among older patients and males, though these did not reach statistical significance (HR = 1.01, 95% CI: 0.97–1.05, *p* = 0.573; HR = 1.64, 95% CI: 0.92–2.90, *p* = 0.093, respectively).

## 4. Discussion

This study provides comprehensive insights into mortality factors among elderly patients with femoral neck fractures treated with arthroplasty, revealing how several variables influence mortality at different postoperative intervals. Our findings are compared with previously discussed studies to elucidate broader trends in this patient demographic.

### 4.1. Demographic and Lifestyle Factors

Age was a prominent factor, with older patients demonstrating significantly higher mortality rates, particularly notable at one year postoperatively in patients with average age 86.86 ± 7.09 (*p* = 0.006). This is consistent with findings from previous research, which has shown that increased age, with a cut off >80 years old in a study by Major et al. [12] and >85 in a study by Meessen et al. [13], is associated with higher postoperative mortality. The bivariate analysis showed increasing positive correlation suggesting higher mortality as time progresses postoperatively. In multivariate regression models, age showed hazard ratios of increasing mortality risk among older patients; however, these were not statistically significant.

Gender also emerged as a significant factor, with male patients experiencing higher mortality rates compared to their female counterparts across the 30-day (7.6% vs. 1.8%, *p* = 0.007), 90-day (11% vs. 5.4%, *p* = 0.045), and 1-year (22% vs. 10.8%, *p* = 0.003) postoperative periods. This aligns with earlier studies suggesting that males may have poorer health at the time of fracture and are less likely to recover from postoperative complications, contributing to higher mortality rates [14,15]. In multivariate regression models, male gender showed hazard ratios of increasing mortality risk; however, these were not statistically significant as well.

Other demographic and sociological factors such as smoking, BMI and nutrition did not show any significant effect on mortality in this study.

### 4.2. Preoperative Functional Status

Preoperative functional assessment of the patients was performed using the Parker Mobility Score, which assesses mobility in three different situations [16]. This score, also called New Mobility Score, has been proven to be a valid predictor for rehabilitation potential and functional outcome postoperatively [8]. In his original article, Parker et al. [11] used the Parker Mobility Score, which was proven to significantly predict mortality at 1 year in patients suffering from a hip fracture. In concordance with our study, patients with a lower Parker Mobility Score were significantly associated with increased mortality at 30 days (*p* = 0.04), 90 days (*p* = 0.009), and at 1 year (*p* < 0.001). The bivariate analysis of the Parker Mobility Score reveals a negative correlation, meaning the lower the Parker Mobility Score, the higher the mortality rate with time at 90 days and 1 year postoperatively. In multivariate regression model, the Parker Score was found to be protective, further reinforcing the role of higher functional status in decreasing the risk of mortality (HR = 0.86, 95% CI: 0.76–0.97, *p* = 0.014).

The Charlson Comorbidity Index was developed based on risk factors that predicted 1-year mortality taking into account both the number and the seriousness of comorbid diseases [10]. It is another significant mortality predictor, with higher scores correlating with increased mortality at all assessed points: 30 days (*p* = 0.004), 90 days (*p* = 0.021), and 1 year (*p* < 0.001). This relationship is well-established in the literature, with higher Charlson scores directly correlating with poorer outcomes in hip fracture patients [17]. A bivariate analysis showed positive coefficients with 30-day, 90-day, and 1-year mortality, indicating that as Total Charlson Score increases, mortality increases. A multivariate regression model revealed that the Charlson Score is predictive of mortality with a hazard ratio of 1.23 (HR = 1.23, 95% CI: 1.04–1.47, *p* = 0.018).

The American Society of Anesthesiologists (ASA) [18] score also showed a strong correlation with mortality, with higher scores indicating worse preoperative health status and correlating with higher mortality rates at 30 days (*p* < 0.001), 90 days (*p* < 0.001), and 1 year (*p* < 0.001). This is consistent with previous findings that the ASA score is a reliable predictor of postoperative mortality, as it reflects the patient’s overall physiological status and ability to withstand surgery. The bivariate analysis reinforced the ASA score’s strong correlation with mortality at all intervals (*p* < 0.001) with positive coefficients 0.187, 0.229, and 0.249 concluding that ASA score increases as mortality rate increases. This was further reinforced with multivariate analysis, revealing that patients with ASA score of II and III had a lower hazard ratio than those with ASA IV.

The results of our study support findings in the literature that indicated that the challenging management of anticoagulation in hip fracture surgeries can significantly impact outcomes, particularly increasing the risk of serious bleeding events and subsequent mortality [14]. The use of Clopidogrel significantly increased mortality, with higher rates observed at 30 days (15.8%, *p* = 0.021), 90 days (21.1%, *p* = 0.035), and 1 year (31.6%, *p* = 0.035). Similarly, antivitamin K medication usage was associated with higher mortality at 90 days (11.5%, *p* = 0.043) and 1 year (20.2%, *p* = 0.046) but not at 30 days. Aspirin and NOACs did not show any significant effects on mortality rate in our study.

Patients on immunosuppressants showed an increased risk of mortality, particularly at 90 days (19.2%, *p* = 0.029) and 1 year (34.6%, *p* = 0.006). Total hip arthroplasty in patients with solid organ transplants was associated with similar incidences of surgical complications compared to controls, and there was a significantly greater incidence of cardiac complications, pneumonia and acute kidney injury [19]. Consequently, these postoperative complications might be the cause of the increased mortality incidence in patients taking immunosuppressant drugs.

### 4.3. Perioperative Characteristics

Despite earlier studies revealing an increased risk of mortality with surgical delay in hip fracture patients [20,21], delay to surgical treatment was not associated with significant increased mortality rate in our study. Bivariate analysis of operative delay as well showed no significant correlation with mortality rate over time. A more recent study by Castellanos et al. [22] revealed increased rates of mortality rate at 30 days with operative delay; however, this increase in mortality was not significant. This correlates with our study where there is an increased mortality rate seen at 30 and 90 days postoperatively without any significant statistical correlation.

On the other hand, prolonged length of hospital stay was associated with increased rate of mortality at 1 year postoperatively only (*p* = 0.038). In contrast, a study conducted by Yoo et al. [23] showed that patients discharged within 10 days after hip surgery have increased mortality rates at 1 year postoperatively. Ek et al. [24] tackled this issue as well and their results, similar to ours, revealed an increased mortality rate with longer hospital stay (>9–12 days) at 4 months postoperatively. In addition, the study mentioned revealed that a lower length of hospital stay was associated with an increased mortality rate when patients were discharged too early. Theoretically, patients with an increased length of hospital stay are subject to an increased risk of nosocomial infections [25], which might explain the increased overall mortality rate. However, patients discharged too early might be subject to postoperative complications as well as readmissions, which might affect the mortality rate as well. Extensive studies and select parameters are needed to surmise the exact effect of length of hospital stay on the mortality rate in patients undergoing hip fracture surgeries.

Different approaches to the hip did not have any significant effect on mortality in our study, adding to previous studies that there is no advantage of a certain approach with regards to mortality rate [26].

The results of this study concluded that patients undergoing hemiarthroplasty had a significantly increased mortality rate compared to patients undergoing total hip arthroplasty at 30 days (*p* = 0.024), 90 days (*p* = 0.005), and 1 year (*p* < 0.001) postoperatively. The results in the literature, however, are contradictory. Many studies in the literature that compared hemiarthroplasty to total hip arthroplasty found no significant difference with regards to mortality rate between these two groups [27,28,29]. In concordance with our study, Peng et al. [30] conducted a meta-analysis comparing outcomes of hemiarthroplasty and total hip arthroplasty. Their results showed a significant difference in mortality at 1 year in favor of total hip arthroplasty. These results were attributed to better functional outcome and better mobility in the total hip arthroplasty group, which was associated with decreased postoperative medical complications such as cardiovascular disease.

Studies in the literature reveal that there is no significant difference in mortality rate between general and spinal anesthesia in patients undergoing hip surgery [31,32,33]. Spinal anesthesia, however, can cause intraoperative hypotension by blocking the sympathetic nervous system [34]. This gave rise to continuous spinal anesthesia that can theoretically decrease intraoperative hypotension by giving small incremental boluses of anesthetics [34]. However, the results in the literature are conflicting and a recent meta-analysis showed that intraoperative hypotension was not a risk factor for increased mortality [35]. The advantage of continuous spinal anesthesia therefore becomes questionable, and in our study, it was associated with a significant increase in mortality. Our results revealed that among the types of anesthesia, continuous spinal anesthesia significantly had the highest mortality rate at 90 days and 1 year with 26.1% and 39.1%, respectively. General and spinal anesthesia had close mortality rates at 90 days of 5.4% and 6.5%, respectively, as well at 1 year postoperatively with 10.7% and 14.9%, respectively.

The National Nosocomial Infections Surveillance (NNIS) score was developed to assess risk factors for surgical site infection perioperatively, and is a score that ranges between 0 and 3, based on the patient’s ASA score, surgery classification as dirty-infected or contaminated, and the length of the operation performed [36,37]. In our study, patients with NNIS score of 1 had significantly higher mortality rates than patients with a score of 0, at 30 days (6.9% vs. 0.5%, respectively, *p* = 0.002), 90 days (13.3% vs. 1.5%, respectively, *p* < 0.001), and at 1 year (22.3% vs. 6.9%, respectively, *p* < 0.001). This is a significant finding and begs consideration when taking into account risk factors for mortality in this population. Patients with a NNIS score of 2 had 0% mortality rate at 30 days, 90 days, and 1 year; however, there were only six patients in this category, and this low population might underestimate the effect of this score on mortality.

In our study, preoperative complications and postoperative surgical complications had no significant effect on mortality rates. In contrast, postoperative medical complications were strongly associated with higher mortality rates seen at 30 days (<0.001), 90 days (*p* = 0.013), and 1 year (*p* = 0.002) postoperatively. Urinary tract infection (3.3%), pneumonia (1.8%), delirium (1.3%), and heart failure (1%) were the most common postoperative medical complications. In multivariate regression model, postoperative medical complications had the strongest predictor of mortality with a hazard ratio of 2.88, implicating an almost three-fold higher risk to mortality among patients (HR = 2.88, 95% CI: 1.56–5.34, *p* < 0.001).

Patients who received a blood transfusion did not exhibit significantly increased mortality rates at 30 (*p* = 0.224) and 90 days (*p* = 0.08) postoperatively. However, at 1 year postop, patients who received a blood transfusion showed a significantly increased mortality rate of 23.9% vs. 10.2% (*p* < 0.001). In concordance with our study, Greenhalgh et al. [38] conducted a retrospective study on 324 patients and found that receiving a blood transfusion increased the absolute mortality risk at 1 year postoperatively by 2.4-fold.

It is important to emphasize that this relationship likely reflects association rather than causation. Sicker patients with higher comorbidity burden are more likely to require transfusion, which may partly explain their higher subsequent mortality risk. Thus, transfusion may serve as a marker of frailty and perioperative instability rather than a direct cause of death.

### 4.4. Survival Rates at 5 and 10 Years

The mortality rate of patients in our study at 5 years and 10 years were 48.36% (survival rate= 51.64%) and 71.03% (survival rate = 28.97%), respectively. The average time to death was 3.3 years.

Various other studies calculated mortality rates of hip fracture patients regardless of the treatment performed. Loh et al. [39] showed 5-year and 10-year mortality rates at 38.9% and 61.6%, respectively. Tiihonen et al. [40] revealed a 10-year mortality rate of 79.8%. Paksima et al. [41] reported a 5-year and 10-year mortality rate of 41.2% and 75.3%, respectively.

In these studies, age and comorbidities were the predominant related risk factors, in concordance with our study. The difference in mortality rates could be related to demographic characteristics, comorbidities, social habits, and the different regional treatment options. Comparison with multicenter prospective cohorts and recent meta-analyses further reinforces our findings, as these larger datasets consistently highlight advanced age, comorbidity burden, and pre-fracture mobility status as the strongest predictors of mortality. Integrating our single-center results with these broader investigations enhances the external validity and generalizability of our conclusions.

### 4.5. Practical Implementation of Risk Stratification Tools

Although the ASA score, Charlson Comorbidity Index, and Parker Mobility Score were adequately analyzed in this study, their true utility lies in their practical application. These tools can be systematically integrated into perioperative protocols to guide decision-making. For example, high ASA or Charlson scores could trigger early involvement of geriatricians and cardiologists, optimization of chronic diseases, and closer postoperative monitoring. Similarly, a low Parker Score could identify patients who would benefit from early physiotherapy, nutritional support, and more intensive discharge planning [8,16]. Embedding these scores into multidisciplinary care pathways allows clinicians to move beyond risk description toward proactive risk modification, a strategy supported by recent orthogeriatric management literature [15,24].

### 4.6. Limitations

This is a single-center study conducted in France, and the findings might not be generalizable in other settings. The study is also retrospective, which might limit causal interpretation and may introduce bias. Unmeasured factors, such as socioeconomic status and frailty, could also influence mortality but were not analyzed in our study.

## 5. Conclusions

There are many factors that can affect postoperative mortality rates in patients undergoing hip arthroplasty. Some of these factors are constant including age, gender, and comorbidities. Other factors can be modified or prevented and those include immunosuppressants, clopidogrel, antivitamin K, continuous spinal anesthesia, hemi-arthroplasty, and postoperative complications. These findings underscore the importance of comprehensive preoperative evaluation, multidisciplinary care, and early intervention to address modifiable risk factors. Implementing targeted protocols, optimizing comorbidity management, careful selection of anesthetic techniques, and proactive prevention of complications could significantly improve survival in this vulnerable patient population. Further research in multicentric prospective studies might be needed to validate these findings and explore additional strategies to optimize survival rate in hip fracture patients.

## Figures and Tables

**Table 1:** Demographic and surgical characteristics of the patients (*N* = 397).

Characteristics		*Mean ± Std* or *N* (%)
**Age**		83.26 ± 10.4
**BMI**		23.09 ± 3.84
**Gender**	Male	118 (29.7%)
	Female	279 (70.3%)
**Smoker (*N* = 384)**	Yes	42 (10.9%)
	No	342 (89.1%)
**Surgical Side**	Right	186 (46.9%)
	Left	211 (53.1%)
**Garden Classification**	I	3 (0.8%)
	II	5 (1.3%)
	III	38 (9.6%)
	IV	351 (88.4%)
**Surgical Approach**	Postero-lateral	335 (84.4%)
	Antero-lateral	53 (13.4%)
	Hardinge	6 (1.5%)
	Hueter	3 (0.8%)
**Hip Replacement**	Total	140 (35.3%)
	Hemiarthroplasty	257 (64.7%)
**Cemented Prosthesis**	Yes	59 (14.9%)
	No	338 (85.1%)

**Table 2:** Distribution of patients according to Total Charlson Score, ASA score, and Parker Mobility Score (*N* = 397).

Total Charlson Score	*N* (%)	ASA Score	*N* (%)	Parker Mobility Score (*N* = 352)	*N* (%)
0	2 (0.5%)	1	40 (10.1%)	0	2 (0.6%)
1	6 (1.5%)	2	168 (42.3%)	1	6 (1.7%)
2	13 (3.3%)	3	170 (42.8%)	2	22 (6.3%)
3	27 (6.8%)	4	19 (4.8%)	3	48 (13.6%)
4	71 (17.9%)			4	57 (16.2%)
5	94 (23.7%)			5	36 (10.2%)
6	75 (18.9%)			6	37 (10.5%)
7	55 (13.9%)			7	26 (7.4%)
8	29 (7.3%)			8	8 (2.3%)
9	20 (5.0%)			9	110 (31.3%)
10	4 (1.0%)				
13	1 (0.3%)				

*ASA = American Society of Anesthesiologists.*

**Table 3:** Pre-op complications.

Complications	*N* (%)
Heart Failure	1 (0.3%)
Anaphylactic Shock	1 (0.3%)
Acute Pulmonary Edema	1 (0.3%)

**Table 4:** Post-op medical complications.

Complications	*N* (%)	Complications	*N* (%)
Acute Coronary Syndrome	1 (0.3%)	Hypoglycemia	1 (0.3%)
Acute Urinary Retention	2 (0.5%)	Melena	1 (0.3%)
Acute Kidney Injury	2 (0.5%)	Oral Mycosis	1 (0.3%)
Anemia	3 (0.8%)	Phlebitis	1 (0.3%)
Atrial Fibrillation	1 (0.3%)	Pneumonia	7 (1.8%)
Cardiopulmonary Arrest	1 (0.3%)	Prostatitis	1 (0.3%)
Delirium	5 (1.3%)	Pulmonary Embolism	2 (0.5%)
Deep Venous Thrombosis	4 (1%)	Respiratory Failure	1 (0.3%)
Esophagitis	1 (0.3%)	Uncontrolled Diabetes	1 (0.3%)
Heart Failure	4 (1%)	Upper Respiratory Infection	1 (0.3%)
Hematoma	1 (0.3%)	Urinary Tract Infection	13 (3.3%)

**Table 5:** Post-op surgical complications.

Complications	*N* (%)	Complications	*N* (%)
Acetabular Implant Loosening	1 (0.3%)	No osteointegration	1 (0.3%)
Bowel Obstruction	1 (0.3%)	Painful Scar	1 (0.3%)
Delayed Wound Healing	1 (0.3%)	Periprosthetic Fracture	8 (2%)
Dislocation	15 (3.8%)	Prosthesis Subsidence	3 (0.8%)
Gluteus Medius Tendinopathy	2 (0.5%)	Quadriceps Muscular Atrophy	1 (0.3%)
Hematoma	6 (1.5%)	Sciatic Nerve Injury	2 (0.5%)
Leg Length Discrepancy	1 (0.3%)	Wound Dehiscence	1 (0.3%)

**Table 6:** Mortality rates according to risk groups.

	Risk Groups			*p*-Value
	Low (*N* = 53)	Medium (*N* = 169)	High (*N* = 175)	
**30 Days Mortality (*N* = 14)**	0 (0%)	1 (0.6%)	13 (7.4%)	**<0.001**
**90 Days Mortality (*N* = 28)**	0 (0%)	5 (3%)	23 (13.1%)	**<0.001**
**1 Year Mortality (*N* = 56)**	0 (0%)	18 (10.7%)	38 (21.7%)	**<0.001**

**Table 7:** Effect of different factors on mortality rates at 30 days, 90 days, and 1 year.

		30 Days Mortality		*p*-Value	90 Days Mortality		*p*-Value	1 Year Mortality		*p*-Value
		No	Yes		No	Yes		No	Yes	
**Age (years)**		83.08 ± 10.48	88.14 ± 5.53	0.064	83 ± 10.56	86.68 ± 7.25	0.099	82.67 ± 10.74	86.86 ± 7.09	**0.006**
**BMI**		23.09 ± 3.83	22.90 ± 4.15	0.992	23.14 ± 3.74	22.32 ± 5.05	0.167	23.20 ± 3.785	22.38 ± 4.136	0.125
**Gender**	**Male**	109 (92.4%)	9 (7.6%)	**0.007**	105 (89%)	13 (11%)	**0.045**	92 (78%)	26 (22%)	**0.003**
	**Female**	274 (98.2%)	5 (1.8%)		264 (94.6%)	15 (5.4%)		249 (89.2%)	30 (10.8%)	
**Smoker**	**No**	329 (96.2%)	13 (3.8%)	1	317 (92.7%)	25 (7.3%)	1	296 (86.5%)	46 (13.5%)	0.164
	**Yes**	41 (97.6%)	1 (2.4%)		39 (92.9%)	3 (7.1%)		33 (78.6%)	9 (21.4%)	
**Hospitalization period (Days)**		9.51 ± 6.32	10.3 ± 4.72	0.696	9.36 ± 5.86	12.20 ± 10.68	0.208	9.275 ± 5.91	11.21 ± 8.11	**0.038**
**OR Delay (hours)**		42.89 ± 69.89	72.39 ± 54.03	0.119	43.26 ± 71.02	52.77 ± 45.98	0.486	43.36 ± 73.21	47.40 ± 41.07	0.687
**Nutritional Status (Proteins)**		69.23 ± 6.52	65.92 ± 6.84	0.055	69.16 ± 6.49	68.5 ± 7.37	0.57	69.13 ± 6.36	69 ± 7.61	0.925
**Aspirin**	**No**	290 (97%)	9 (3%)	1	280 (93.6%)	19 (6.4%)	0.617	262 (87.6%)	37 (12.4%)	0.177
	**Yes**	86 (96.6%)	3 (3.4%)		82 (92.1%)	7 (7.9%)		73 (82%)	16 (18%)	
**Clopidogrel**	**No**	360 (97.3%)	10 (2.7%)	**0.021**	347 (93.8%)	23 (6.2%)	**0.035**	322 (87%)	48 (13%)	**0.035**
	**Yes**	16 (84.2%)	3 (15.8%)		15 (78.9%)	4 (21.1%)		13 (68.4%)	6 (31.6%)	
**Antivitamin K**	**No**	279 (97.2%)	8 (2.8%)	0.214	271 (94.4%)	16 (5.6%)	**0.043**	252 (87.8%)	35 (12.2%)	**0.046**
	**Yes**	98 (94.2%)	6 (5.8%)		92 (88.5%)	12 (11.5%)		83 (79.8%)	21 (20.2%)	
**NOACs**	**No**	371 (96.4%)	14 (3.6%)	1	357 (92.7%)	28 (7.3%)	1	330 (85.7%)	55 (14.3%)	1
	**Yes**	6 (100%)	0 (0%)		6 (100%)	0 (0%)		5 (83.3%)	1 (16.7%)	
**Immunosuppressant**	**No**	358 (97%)	11 (3%)	0.057	346 (93.8%)	23 (6.2%)	**0.029**	322 (87.3%)	47 (12.7%)	**0.006**
	**Yes**	23 (88.5%)	3 (11.5%)		21 (80.8%)	5 (19.2%)		17 (65.4%)	9 (34.6%)	
**Parker Mobility Score**		5.897 ± 2.575	4.583 ± 1.928	**0.04**	5.929 ± 2.594	4.791 ± 1.864	**0.009**	6.053 ± 2.598	4.666 ± 2.006	**<0.001**
**Total Charlson Score**		5.41 ± 1.875	6.86 ± 1.46	**0.004**	5.398 ± 1.889	6.25 ± 1.554	**0.021**	5.263 ± 1.816	6.642 ± 1.833	**<0.001**
**NNIS Score**	**0**	202 (99.5%)	1 (0.5%)	**0.002**	200 (98.5%)	3 (1.5%)	**<0.001**	189 (93.1%)	14 (6.9%)	**<0.001**
	**1**	175 (93.1%)	13 (6.9%)		163 (86.7%)	25 (13.3%)		146 (77.7%)	42 (22.3%)	
	**2**	6 (100.0%)	0 (0%)		6 (100.0%)	0 (0.0%)		6 (100.0%)	0 (0.0%)	
**ASA Score**	**1**	40 (100%)	0 (0%)	**<0.001**	40 (100%)	0 (0%)	**<0.001**	40 (100%)	0 (0%)	**<0.001**
	**2**	167 (99.4%)	1 (0.6%)		165 (98.2%)	3 (1.8%)		154 (91.7%)	14 (8.3%)	
	**3**	160 (94.1%)	10 (5.9%)		149 (87.6%)	21 (12.4%)		135 (79.4%)	35 (20.6%)	
	**4**	16 (84.2%)	3 (15.8%)		15 (78.9%)	4 (21.1%)		12 (63.2%)	7 (36.8%)	
**pRBCs Transfusion**	**No**	276 (97.2%)	8 (2.8%)	0.236	268 (94.4%)	16 (5.6%)	0.08	255 (89.8%)	29 (10.2%)	**<0.001**
	**Yes**	107 (94.7%)	6 (5.3%)		101 (89.4%)	12 (10.6%)		86 (76.1%)	27 (23.9%)	
**Surgical Side**	**Right**	179 (96.2%)	7 (3.8%)	0.81	174 (93.5%)	12 (6.5%)	0.66	165 (88.7%)	21 (11.3%)	0.13
	**Left**	204 (96.7%)	7 (3.3%)		195 (92.4%)	16 (7.6%)		176 (83.4%)	35 (16.6%)	
**Garden Classification**	**I**	3 (100%)	0 (0%)	0.513	3 (100%)	0 (0%)	0.676	3 (100%)	0 (0%)	0.783
	**II**	4 (80%)	1 (20%)		4 (80%)	1 (20%)		4 (80%)	1 (20%)	
	**III**	37 (97.4%)	1 (2.6%)		36 (94.7%)	2 (5.3%)		33 (86.8%)	5 (13.2%)	
	**IV**	339 (96.6%)	12 (3.4%)		326 (92.9%)	25 (7.1%)		301 (85.8%)	50 (14.2%)	
**Type of hip arthroplasty**	**HA**	244 (94.9%)	13 (5.1%)	**0.024**	232 (90.3%)	25 (9.7%)	**0.005**	209 (81.3%)	48 (18.7%)	**<0.001**
	**THA**	139 (99.3%)	1 (0.7%)		137 (97.9%)	3 (2.1%)		132 (94.3%)	8 (5.7%)	
**Approach**	**PL**	323 (96.4%)	12 (3.6%)	0.883	311 (92.8%)	24 (7.2%)	0.743	292 (87.2%)	43 (12.8%)	0.243
	**AL**	51 (96.2%)	2 (3.8%)		50 (94.3%)	3 (5.7%)		41 (77.4%)	12 (22.6%)	
	**Hardinge**	6 (100%)	0 (0%)		5 (83.3%)	1 (16.7%)		5 (83.3%)	1 (16.7%)	
	**Hueter**	3 (100%)	0 (0%)		3 (100%)	0 (0%)		3 (100%)	0 (0%)	
**Cemented**	**No**	325 (96.2%)	13 (3.8%)	0.703	313 (92.6%)	25 (7.4%)	0.782	287 (84.9%)	51 (15.1%)	0.178
	**Yes**	58 (98.3%)	1 (1.7%)		56 (94.9%)	3 (5.1%)		54 (91.5%)	5 (8.5%)	
**Anesthesia**	**GA**	200 (97.6%)	5 (2.4%)	0.207	194 (94.6%)	11 (5.4%)	**0.028**	183 (89.3%)	22 (10.7%)	**0.011**
	**SSS**	162 (96.4%)	6 (3.6%)		157 (93.5%)	11 (6.5%)		143 (85.1%)	25 (14.9%)	
	**CS**	20 (87%)	3 (13%)		17 (73.9%)	6 (26.1%)		14 (60.9%)	9 (39.1%)	
	**Regional**	1 (100%)	0 (0%)		1 (100%)	0 (0%)		1 (100%)	0 (0%)	
**Complications pre-op**	**No**	380 (96.4%)	14 (3.6%)	1	367 (93.1%)	27 (6.9%)	0.197	339 (86%)	55 (14%)	0.367
	**Yes**	3 (100%)	0 (0%)		2 (66.7%)	1 (33.3%)		2 (66.7%)	1 (33.3%)	
**Medical Complications post-op**	**No**	326 (98.2%)	6 (1.8%)	**<0.001**	314 (94.6%)	18 (5.4%)	**0.013**	293 (88.3%)	39 (11.7%)	**0.002**
	**Yes**	57 (87.7%)	8 (12.3%)		55 (84.6%)	10 (15.4%)		48 (73.8%)	17 (26.2%)	
**Surgical Complications post-op**	**No**	337 (96.3%)	13 (3.7%)	1	327 (93.4%)	23 (6.6%)	0.356	302 (86.3%)	48 (13.7%)	0.541
	**Yes**	46 (97.9%)	1 (2.1%)		42 (89.4%)	5 (10.6%)		39 (83%)	8 (17%)	

*BMI = body mass index; NOACs = New Oral Anticoagulants; ASA = American Society of Anesthesiologists; HA = hemiarthroplasty; THA = total hip arthroplasty; PL = Postero-lateral; AL = Antero-Lateral; GA = General Anesthesia; SSS = Single Shot Spinal; CS = Continuous Spinal. Bolded p-values indicate statistical significance*

**Table 8:** Bivariate correlations for mortality with several patient characteristics.

	Age	BMI	OR Delay (hours)	Hospitalization Period (Days)	Nutritional Status (Proteins)	Parker Mobility Score	Total Charlson Score	ASA score
**30 Days Mortality**	0.090	−0.009	0.078	0.020	−0.095	−0.093	0.142 **	0.187 ***
**90 Days Mortality**	0.091	−0.053	0.035	0.109 *	−0.026	−0.112 *	0.116 *	0.229 ***
**1 Year Mortality**	0.140 **	−0.075	0.020	0.105 *	−0.007	−0.190 ***	0.256 ***	0.249 ***

*BMI = body mass index; ASA = American Society of Anesthesiologists;* * *= p <* 0.05; ** *= p <* 0.01; *** *= p <* 0.001. *Bolded p-values indicate statistical significance*

**Table 9:** Multivariate Cox proportional hazards regression model to predict mortality at 1-year post-op.

Variables	Hazard Ratio	95% CI for HR	*p*-Value
Age (years)	1.01	0.97–1.05	0.573
Gender (Ref: Female)	1.64	0.92–2.90	0.093
ASA score (Ref: ASA IV)			
ASA I	0.00	0.00–3.04 × 10^3^	0.970
ASA II	0.30	0.11–0.81	**0.017**
ASA III	0.40	0.17–0.95	**0.038**
Charlson Comorbidity Index	1.23	1.04–1.47	**0.018**
Parker Mobility Score	0.86	0.76–0.97	**0.014**
Post-op Medical Complication (Ref: No Complication)	2.88	1.56–5.34	**<0.001**

*ASA Score = American Society of Anesthesiologists Score; HR = hazard ratio.*

## Data Availability

The datasets presented in this article are not readily available because of confidentiality restrictions.
